# Lipotoxic Stress Induces Pancreatic *β*-Cell Apoptosis through Modulation of Bcl-2 Proteins by the Ubiquitin-Proteasome System

**DOI:** 10.1155/2015/280615

**Published:** 2015-05-06

**Authors:** Sara A. Litwak, Jibran A. Wali, Evan G. Pappas, Hamdi Saadi, William J. Stanley, L. Chitra Varanasi, Thomas W. H. Kay, Helen E. Thomas, Esteban N. Gurzov

**Affiliations:** ^1^St Vincent's Institute of Medical Research, Melbourne, VIC 3065, Australia; ^2^Department of Medicine, St. Vincent's Hospital, The University of Melbourne, Melbourne, VIC 3065, Australia

## Abstract

Pancreatic *β*-cell loss induced by saturated free fatty acids (FFAs) is believed to contribute to type 2 diabetes. Previous studies have shown induction of endoplasmic reticulum (ER) stress, increased ubiquitinated proteins, and deregulation of the Bcl-2 family in the pancreas of type 2 diabetic patients. However, the precise mechanism of *β*-cell death remains unknown. In the present study we demonstrate that the FFA palmitate blocks the ubiquitin-proteasome system (UPS) and causes apoptosis through induction of ER stress and deregulation of Bcl-2 proteins. We found that palmitate and the proteasome inhibitor MG132 induced ER stress in *β*-cells, resulting in decreased expression of the prosurvival proteins Bcl-2, Mcl-1, and Bcl-XL, and upregulation of the prodeath BH3-only protein PUMA. On the other hand, pharmacological activation of the UPS by sulforaphane ameliorated ER stress, upregulated prosurvival Bcl-2 proteins, and protected *β*-cells from FFA-induced cell death. Furthermore, transgenic overexpression of Bcl-2 protected islets from FFA-induced cell death *in vitro* and improved glucose-induced insulin secretion *in vivo*. Together our results suggest that targeting the UPS and Bcl-2 protein expression may be a valuable strategy to prevent *β*-cell demise in type 2 diabetes.

## 1. Introduction

The prevalence of type 2 diabetes has doubled in the past 30 years and currently affects 360 million people worldwide [1]. The consumption of a diet rich in saturated free fatty acids (FFAs) is one of the main environmental causes of obesity and subsequent type 2 diabetes development [2]. In obese humans, there is a high demand for insulin production by pancreatic *β*-cells to maintain normal blood glucose levels. As a result, *β*-cell function and survival are adversely affected. Compared to pancreases of nondiabetic individuals, diabetic subjects had a decrease in both *β*-cell mass and insulin levels [3–6]. *β*-cells have a relatively low regenerative potential [7], and therefore pathogenic insults upon them are likely to have detrimental effects on glucose homeostasis [5]. Chronic exposure to saturated FFAs causes *β*-cell loss (lipotoxicity) and may contribute to type 2 diabetes [2]. The molecular mechanism, however, remains poorly understood [8].

The ubiquitin-proteasome system (UPS) is part of the physiological unfolded protein response, which ameliorates endoplasmic reticulum (ER) stress [9]. The UPS degrades misfolded or damaged proteins to maintain cellular homeostasis. Previous studies have demonstrated increased levels of ubiquitinated proteins in islets obtained from type 2 diabetic subjects [10, 11]. The high levels of protein ubiquitination correlate with *β*-cell death [10]. Whether the observed increase in protein ubiquitination and *β*-cell apoptosis is secondary to inactivation of the UPS is still unknown.

The intrinsic pathway of apoptosis is controlled by the Bcl-2 family of proteins present in the ER, mitochondria, and nuclear membrane of the cells [12]. They share similar structures and are generally characterized by the presence of short conserved sequences of up to 20 residues called Bcl-2 homology (BH) motifs [13]. There are three types of Bcl-2 proteins: prosurvival, proapoptotic Bcl-2 homology 3- (BH3-) only proteins and downstream multidomain proapoptotic effectors [12, 14].* In vitro* studies have demonstrated that exposure to conditions observed in type 2 diabetes (i.e., high concentrations of saturated FFAs or glucose) causes an imbalance between proapoptotic and prosurvival Bcl-2 proteins in *β*-cells leading to apoptosis [14–16].

Because of the correlation of increased protein ubiquitination and *β*-cell loss in type 2 diabetes, as well as the previous discovery that lipotoxicity induces apoptosis in *β*-cells, we sought to determine whether UPS inactivation by FFAs constitutes the upstream signalling pathway of *β*-cell death. We found that the saturated FFA palmitate inhibits the UPS activity, generating an increase in ubiquitinated protein levels, a build-up of ER stress, and an imbalance in the expression of Bcl-2 protein family members, culminating in *β*-cell apoptosis.

## 2. Materials and Methods

### 2.1. Mice

We maintained mice on a 12 h light-dark cycle in a temperature-controlled room with free access to food and water. PUMA −/− mice were generated in C57BL/6 background as previously described [17]. RIP-Bcl-2 transgenic mice overexpress Bcl-2 in *β*-cells and have been described previously [18]. Bcl-2 overexpression has no obvious consequences for the development and function of the *β*-cells [18]. Mice were fed a high fat diet (Fat Sweden Rodent Diet; Specialty Feeds, Perth, Western Australia) with nutritional composition of 17.6% (w/w) protein and 27% (w/w) fat. The fat composition was 14.59% saturated fat, 11.66% monounsaturated fat, and 0.53% polyunsaturated fat. Mice were maintained at St Vincent's Institute, and experiments were approved by the institutional animal ethics committee.

### 2.2. Culture Conditions for Human and Mouse Islets and MIN6 Cells

Human pancreata were obtained, with informed consent from next-of-kin, from heart-beating, brain-dead donors by the Australian Islet Transplant Consortium. The project was approved by the human ethics committees of the hospitals involved and the Australian Red Cross. Human islets were isolated as described previously [19]. Description of human organ donors is provided in Supplementary Table 1 (in Supplementary Material available online at http://dx.doi.org/10.1155/2015/280615).

Mouse islets of Langerhans were isolated using collagenase P (Roche, Basel, Switzerland) and Histopaque-1077 density gradients (Sigma, St. Louis, MO, USA) as previously described [20]. Islets were washed, hand-picked, and cultured overnight at 37°C in 5% CO_2_ in CMRL medium-1066 (Invitrogen) supplemented with 100 U/mL penicillin, 100 *μ*g/mL streptomycin, 2 mmol/L glutamine, and 10% FCS (JRH Biosciences, Lenexa, KS, USA).

MIN6 cells were a kind gift from Dr. Jun-ichi Miyazaki and were cultured in DMEM medium (Invitrogen) supplemented with 10% FCS as described [21].

### 2.3. Treatments

For FFA exposure, mouse islets and MIN6 cells were cultured in medium with 1% FBS and 1% BSA [22]. Palmitate (Sigma) was dissolved in 90% ethanol [23] and used at a final concentration of 0.5 mM in the presence of 1% BSA resulting in unbound FFA concentrations in the nM range [22]. The proteasome inhibitor MG132 (Sigma) was used at 10 *μ*M; the antioxidant sulforaphane (SFN [24]) was used at 10 *μ*M and added 2 h before the introduction of palmitate to the culture medium. MG132 and SFN were dissolved in dimethyl sulfoxide (Sigma), and controls contained an equal volume of solvents.

### 2.4. Proteasome Activity

Following FFA or MG132 treatment, MIN6 cell lysates (prepared in 0.5% Nonidet P-40 in PBS) were centrifuged at 10,000 g for 10 min at 4°C to clear nuclei and nonlysed cell debris. The proteasome activity in the cleared lysates was determined using a Proteasome Activity Assay Kit (BioVision, CA; catalog number K245-100) according to the manufacturer's instructions. Briefly, 7-amino-4-methylcoumarin standards, positive controls, and samples diluted in assay buffer were loaded in duplicate into a 96-well plate. The fluorescence activity for each sample was measured on an EnSpire multimode plate reader (PerkinElmer) over a 60-minute period using excitation and emission wavelengths of 350 and 445 nm, respectively. For each sample, the proteasome activity was determined based on the standard curve.

### 2.5. Immunohistochemistry and Immunofluorescence

Human and mouse pancreata were fixed in 4% formalin solution at 4°C and embedded in paraffin. Ubiquitin detection by immunohistochemistry was performed by indirect immunoperoxidase staining on 5 *µ*m sections using rabbit anti-ubiquitin (1 : 250, Cell Signaling, Danvers, MA, USA) overnight at 4°C. The next day, sections were washed and incubated with anti-rabbit SP-conjugated secondary antibody followed by ABC reagent contained in the Vectastain Elite ABC Kit (Vector Labs, Burlingame, CA, USA), as per kit instructions. The signal was developed by NiSO_4_/DAB solution.

Immunofluorescence detection of insulin was performed using standard procedures. Briefly, paraffin sections were incubated with guinea pig anti-insulin (1 : 200; DAKO, Denmark) for 1 h. Sections were washed and stained with the secondary antibody Alexa Fluor 488-conjugated goat anti-guinea pig IgG (1 : 200; Life Technologies). Sections were mounted in DAKO fluorescence mounting media and examined by confocal microscopy.

### 2.6. Real-Time PCR

RNA was prepared using the NucleoSpin RNA XS (Macherey-Nagel, Düren, Germany). First-strand cDNA was prepared from 600 ng RNA using the High Capacity cDNA Reverse Transcription kit (Applied Biosystems, Foster City, CA, USA). cDNA was diluted (1 : 20) and real-time PCR was performed using the Rotor-Gene RG-3000 machine (Corbett Research; Qiagen, Hilden, Germany) and the TaqMan PCR Master Mix (AmpliTaq Gold with GeneAmp kit; Applied Biosystems) in 20 *μ*L reaction volumes. Data analyses were performed with the ddCT method using *β*-actin as an internal control. Results are represented as fold induction compared to control. TaqMan gene expression probes for mouse genes (Applied Biosystems) are provided in Supplementary Table 2.

### 2.7. Western Blot

After cell culture and treatment, cells were lysed using RIPA buffer and total proteins were extracted and resolved by SDS-PAGE, transferred onto a nitrocellulose membrane, and immunoblotted with the antibodies indicated in Supplementary Table 3. The intensity values for the proteins were corrected by the values of the housekeeping protein *β*-actin and are shown as fold induction versus the control sample (considered to be 1).

### 2.8. Assessment of Cell Viability

Human islet preparations were assessed for *β*-cell proportion with 10 *μ*m Newport green staining (Molecular Probes, Invitrogen, Grand Island, NY, USA) and analysed by flow cytometry following the method of Ichii et al. [25]. Mouse islets were dispersed into single cells with trypsin. DNA fragmentation was analysed by staining with propidium iodide (PI) as previously described [26]. The percentage of cell death of MIN6 cells was determined in at least 600 cells per experimental condition by inverted fluorescence microscopy after staining with the DNA dyes Hoechst-33342 (HO, 10 *µ*g/mL) and PI (5 *µ*g/mL).

### 2.9. Intravenous Glucose Tolerance Test

Intravenous glucose tolerance test (IV-GTT) was performed in wild-type mice and RIP-Bcl-2 mice fed a high fat diet for 14 weeks after 6 h of fasting according to previously described methods [27]. Briefly, 1 g/kg glucose was injected and plasma samples were obtained at 0, 2, 5, 10, 15, and 30 min to measure insulin concentration by ELISA (Mercodia, Uppsala, Sweden).

### 2.10. Statistical Analysis

Comparisons between groups were made by paired *t*-test or by ANOVA followed by Bonferroni correction. A *P* value < 0.05 was considered statistically significant.

## 3. Results

### 3.1. Ubiquitination Is Enhanced in Pancreatic Islets from Obese Humans and from Mice Fed a High Fat Diet

**Figure 1:**
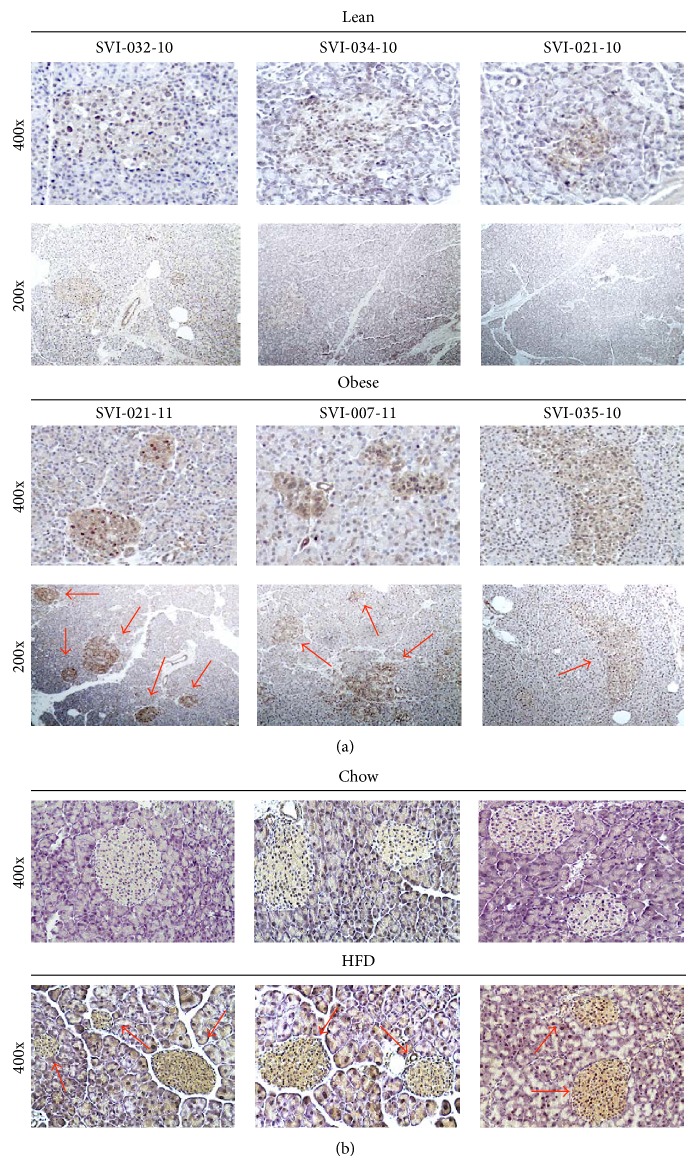
*Immunohistological images of pancreas slides stained for ubiquitin in lean and obese humans and chow and high fat fed mice after 24 weeks.* (a) Pancreases were obtained from human organ donors and then were embedded in paraffin and cut into 5 *µ*m thick sections. Immunohistochemistry was then performed, staining for ubiquitin. (b) Pancreatic sections from mice fed chow or on high fat diet for 24 weeks were stained for ubiquitin. Red arrows indicate islets with higher levels of ubiquitinated protein ((a) and (b)).

To examine the effect of obesity on the UPS in islets, we stained pancreatic sections of lean and obese [body mass index (BMI) > 30 kg/m^2^] humans with anti-ubiquitin antibody. In obese human samples, there was an increased prevalence of ubiquitinated proteins in pancreatic islets when compared to lean controls (Figure 1(a), Supplementary Table 1). Similar results were obtained in pancreas from mice fed a high fat diet for 24 weeks (Figure 1(b)).

**Figure 2:**
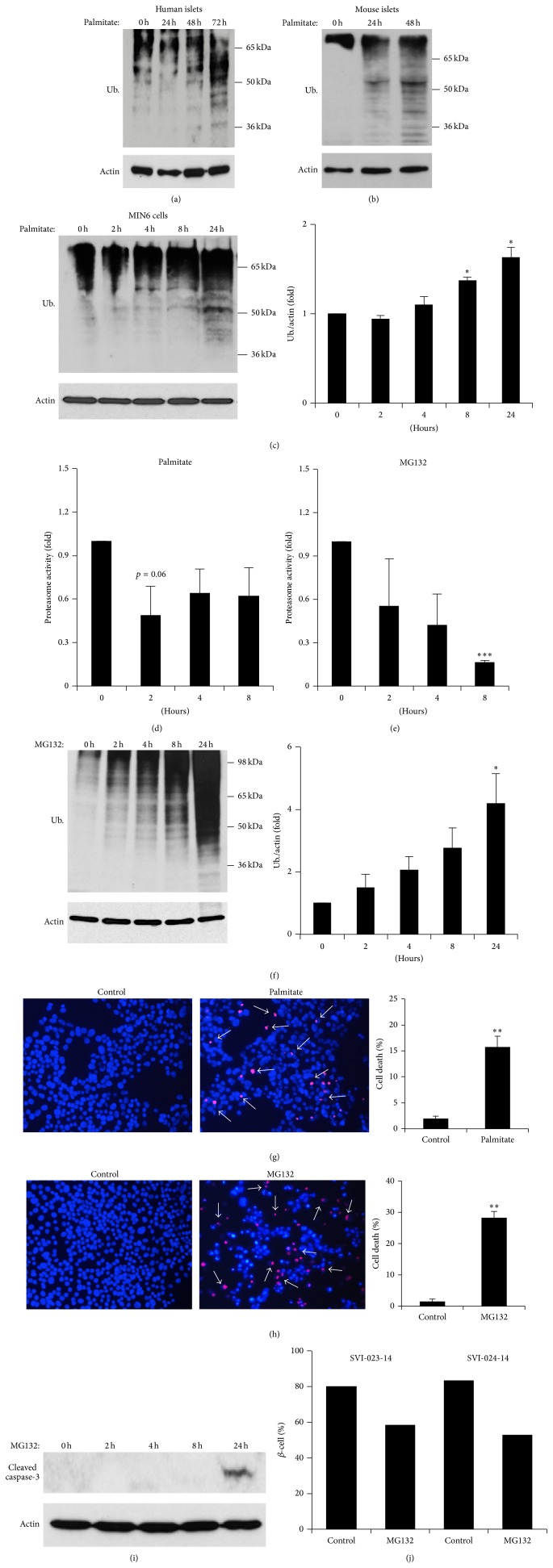
*Palmitate and MG132 treatment induces a similar increase in levels of ubiquitinated proteins in pancreatic islets and β-cells.* ((a)–(c)) Expression of ubiquitinated proteins measured by Western blot in human islets (a), mouse islets (b), or MIN6 cells (c) under control conditions or following 0.5 mM palmitate treatment as indicated. Band intensities were quantified, with values of the specific time points standardised to *β*-actin loading control levels. Results represent fold induction levels, relative to control. ((d)-(e)) MIN6 cells were treated with 0.5 mM palmitate (d) and 10 *μ*M MG132 (e) for 0 (untreated), 2, 4, and 8 h. Proteasome activity was measured using the Proteasome 20S Activity Assay kit. Results represent fold induction levels relative to the control. (f) MIN6 cells were treated with 10 *μ*M MG132 as indicated and levels of ubiquitinated proteins were measured by Western blot analysis. ((g)-(h)) MIN6 cells were treated with 0.5 mM palmitate (g) and 10 *μ*M MG132 (h) for 24 h and cell death was visualised by HO/PI staining. White arrows indicate cell death. (i) Western blot for cleaved caspase-3 in MIN6 cells after treatment with MG132 as indicated. (j) Human islets isolated from organ donors were treated for 24 h with MG132 and % of *β*-cells detected by Newport green staining and flow cytometry. Results are the means ± SEM of 3-4 independent experiments, except for Figures 2(a), 2(b), 2(i), and 2(j) (data representative of 2 independent experiments). ^∗^ *P* < 0.05; ^∗∗^ *P* < 0.01.

To determine whether exposure to increased FFAs had a role in the increased ubiquitin staining observed in obese subjects, human islets were treated with palmitate and levels of ubiquitinated proteins were measured by Western blot analysis. There was a significant increase in the levels of ubiquitinated proteins after palmitate treatment (Figure 2(a)). Similar results were obtained in mouse islets (Figure 2(b)) and MIN6 cells (Figure 2(c)). When quantified, levels of ubiquitinated proteins were significantly increased after 8 h of palmitate treatment. Treatment of MIN6 cells with unsaturated FFAs (oleate) did not increase ubiquitination (data not shown). Direct measurements of proteasome activity in MIN6 cells showed a trend towards decreased activity after palmitate exposure (Figure 2(d)) and a marked reduction in activity after treatment with the synthetic proteasome inhibitor MG132 (Figure 2(e)). As expected, MG132 induced a significant increase in ubiquitinated proteins in MIN6 cells (Figure 2(f)). Next, we examined the effects of proteasome inactivation upon *β*-cell survival by HO/PI. After 24 h of either palmitate or MG132 treatment, there was a significant increase in cell death (Figures 2(g) and 2(h)). In line with these findings, increased activation of caspase-3 was observed, demonstrating apoptosis induction by MG132 (Figure 2(i)). Importantly, we observed a reduction in the proportion of *β*-cells measured by Newport green staining [25] after human islets from two different donors were treated with MG132, suggesting a direct effect of proteasome inhibition on the insulin producing cells (Figure 2(j)).

### 3.2. Proteasome Inactivation Triggers ER Stress and Induces an Imbalance of Bcl-2 Proteins

**Figure 3:**
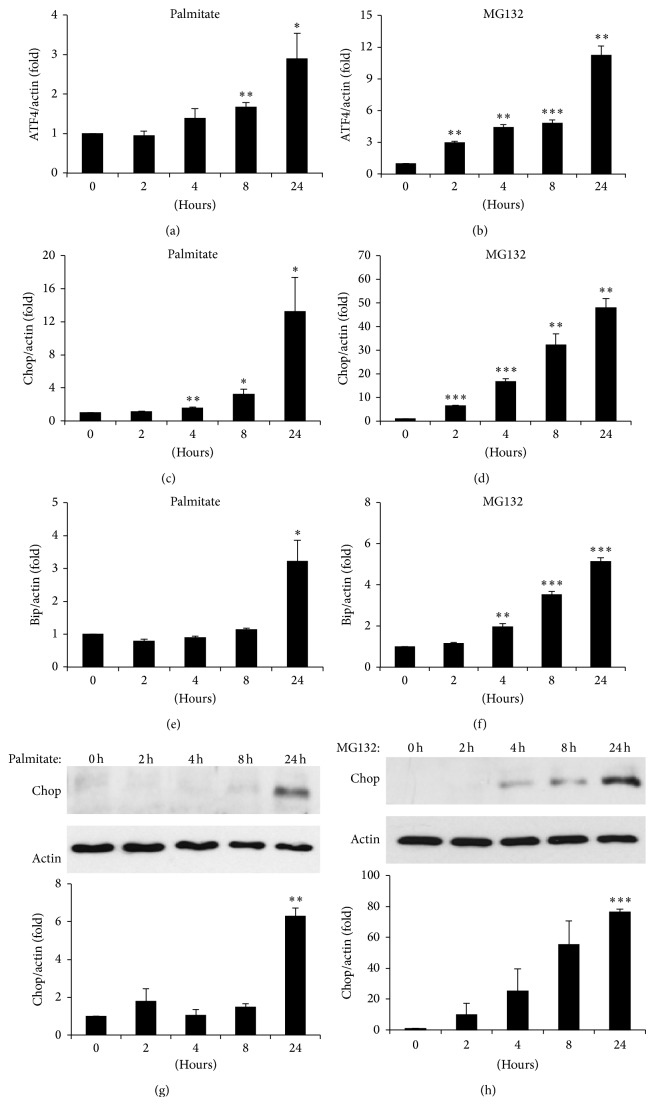
*UPS inactivation induces ER stress in β-cells.* ((a)–(f)) MIN6 cells were treated with 0.5 mM palmitate ((a), (c), and (e)) or 10 *μ*M MG132 ((b), (d), and (f)) and ER stress markers (ATF4, Chop, and Bip) measured as indicated. RNA was isolated using NucleoSpin RNA XS kit and cDNA was made with a High Capacity cDNA Reverse Transcriptase Kit. mRNA was analysed via real-time PCR analysis and standardised to *β*-actin internal control levels. ((g)-(h)) MIN6 cells were treated with 0.5 mM palmitate (g) or 10 *μ*M MG132 (h) as indicated, and levels of Chop protein expression were measured by Western blot. Band intensities were quantified and standardised to *β*-actin loading control. Results are the means ± SEM of 3–5 independent experiments. ^∗^ *P* < 0.05, ^∗∗^ *P* < 0.01, and ^∗∗∗^ *P* < 0.001.

Previous studies suggested that *β*-cells are particularly sensitive to ER stress and this may contribute to palmitate-induced apoptosis [16, 28]. In order to determine the mechanism of *β*-cell death mediated by UPS inactivation, ER stress markers were examined in MIN6 cells. In line with previous studies, palmitate treatment increased mRNA expression of the ER stress markers ATF4, Bip, and Chop (Figures 3(a), 3(c), 3(e), and 3(g)). Similarly, proteasome inactivation by MG132 induced ATF4, Bip, and Chop in MIN6 cells (Figures 3(b), 3(d), 3(f), and 3(h)). Thus, inhibition of the proteasome triggers ER stress and increases expression of various markers in the unfolded protein response pathway.

**Figure 4:**
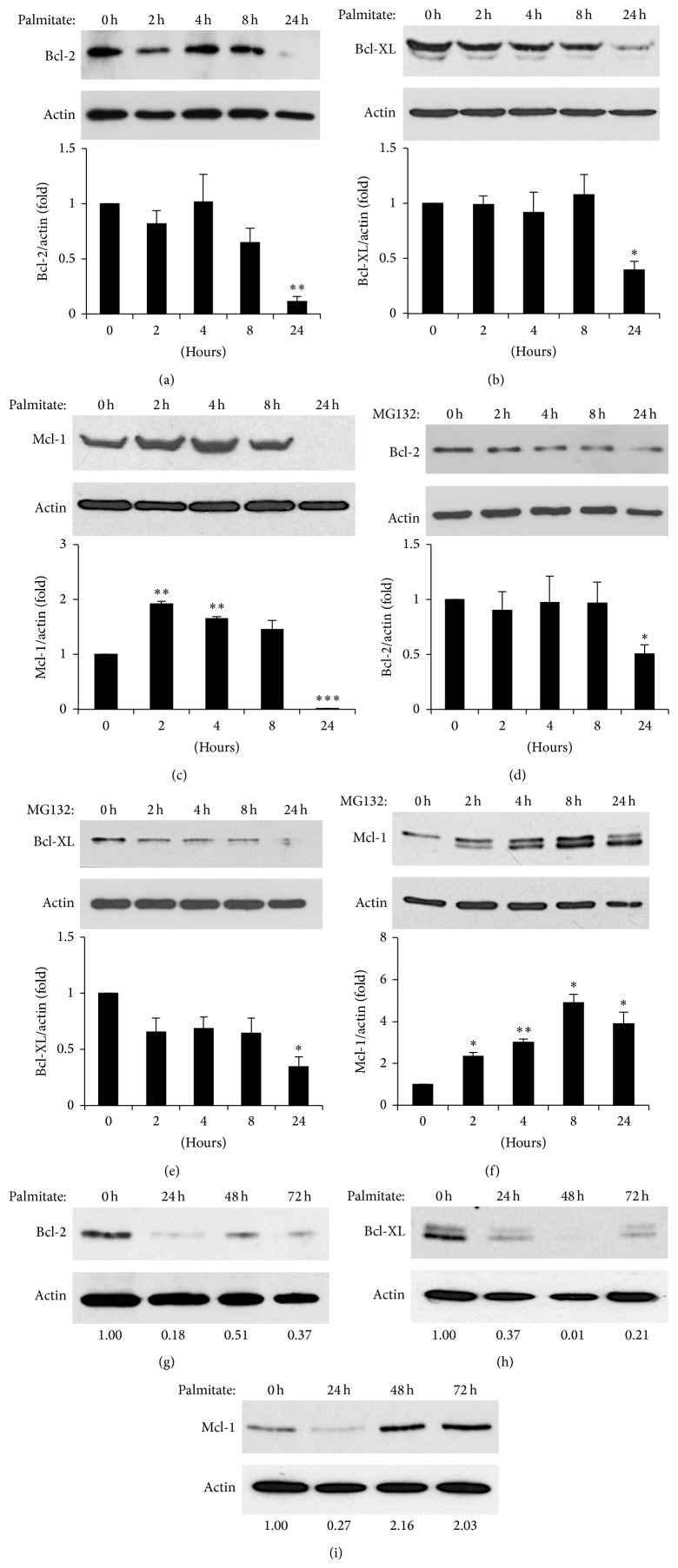
*Palmitate and MG132 decrease levels of prosurvival proteins in pancreatic islets and β-cells.* ((a)–(c)) MIN6 cells were treated with 0.5 mM palmitate as indicated and expression of prosurvival proteins Bcl-2 (a), Bcl-XL (b), and Mcl-1 (c) was measured by Western blot. Band intensities were quantified and values of the specific time points standardised to *β*-actin loading controls. Results are the means ± SEM of 3 independent experiments. ^∗^ *P* < 0.05, ^∗∗^ *P* < 0.01, and ^∗∗∗^ *P* < 0.001. ((d)-(e)) MIN6 cells were treated with 10 *μ*M MG132 as indicated and expression of prosurvival proteins Bcl-2 (d), Bcl-XL (e), and Mcl-1 (f) was measured by Western blot. Band intensities were quantified and values of the specific time points standardised to *β*-actin loading controls. Results are the means ± SEM of 3 independent experiments. ^∗^ *P* < 0.05; ^∗∗^ *P* < 0.01. ((g)–(i)) Expression of prosurvival Bcl-2 proteins in mouse islets after palmitate treatment. Cell lysates were subjected to Western blotting with antibodies detecting Bcl-2 (g), Bcl-XL (h), and Mcl-1 (i). Quantification of protein bands is indicated at the bottom as a ratio to *β*-actin loading control. The results are representative of two independent experiments.

ER stress affects the expression of Bcl-2 proteins in pancreatic *β*-cells [14, 16]. Thus, we investigated whether these proteins are involved in UPS-mediated *β*-cell death. Both palmitate and MG132 treatment resulted in a marked reduction in the levels of prosurvival Bcl-2 proteins in MIN6 cells (Figure 4). After 24 h of palmitate or MG132 treatment, there was a downregulation of Bcl-2 (Figures 4(a) and 4(d)) and Bcl-XL (Figures 4(b) and 4(e)) protein expression. Interestingly, there was a significant increase at 2 h in levels of Mcl-1 protein expression preceding the decline after 4 h of palmitate treatment (Figure 4(c)). This phenomenon was also observed in MG132 treated cells in addition to a second Mcl-1 protein band suggesting posttranscriptional modulation (Figure 4(f)). Nonetheless, prolonged exposure to palmitate and MG132 induced a general reduction of prosurvival Bcl-2 proteins. Similar results were obtained in isolated mouse islets exposed to palmitate (Figures 4(g)–4(i)). In contrast to the reduced levels of protein, mRNA expression of these prosurvival factors was either increased or not modified with palmitate or MG132 treatments (Supplementary Figures 1(a)–1(d)). Taken together, these results indicate a posttranscriptional modulation of antiapoptotic Bcl-2 proteins by UPS inactivation.

**Figure 5:**
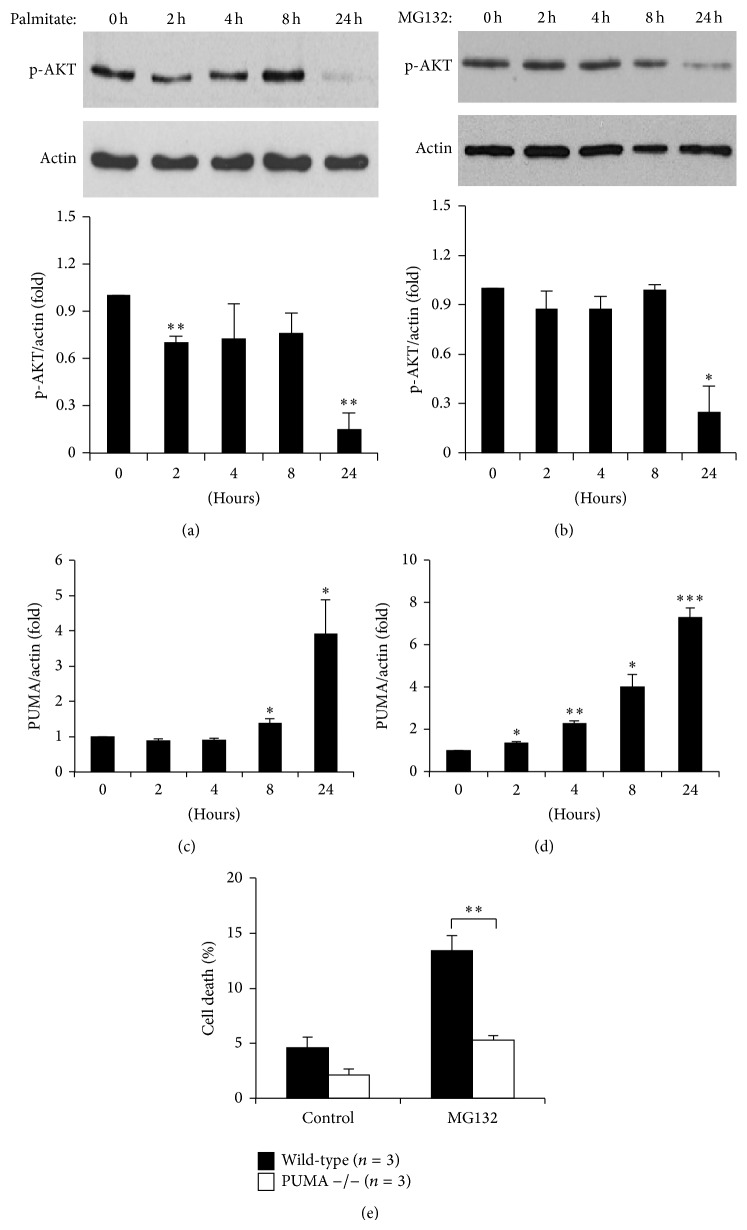
*Palmitate and MG132 activate AKT and PUMA in pancreatic β-cells.* ((a)-(b)) MIN6 cells were treated with 0.5 mM palmitate (a) or 10 *μ*M MG132 (b) as indicated and expression of p-AKT was measured by Western blot. Band intensities were quantified and values of the specific time points standardised to *β*-actin loading controls. ((c)-(d)) MIN6 cells were treated with 0.5 mM palmitate (c) or 10 *μ*M MG132 (d) and expression of PUMA was measured by qPCR. (e) Islets were obtained from wild-type C57BL/6 and PUMA −/− mice and treated with 10 *μ*M MG132 for 24 h and subsequent levels of cell death were determined by FACS analysis. Results are the means ± SEM of 3–5 independent experiments. ^∗^ *P* < 0.05, ^∗∗^ *P* < 0.01, and ^∗∗∗^ *P* < 0.001.

The proapoptotic BH3-only protein PUMA plays an important role in palmitate-induced *β*-cell apoptosis [16]. Phosphorylation of AKT, which downregulates PUMA in control conditions [16], was inhibited after palmitate and MG132 treatment (Figures 5(a) and 5(b)). In agreement, palmitate and MG132 increased PUMA mRNA expression (Figures 5(c)-5(d)). Importantly, islets lacking PUMA expression were protected against UPS inactivation by MG132 (Figure 5(e)).

### 3.3. SFN Protects *β*-Cells from UPS Inactivation and Palmitate-Induced Cell Death

**Figure 6:**
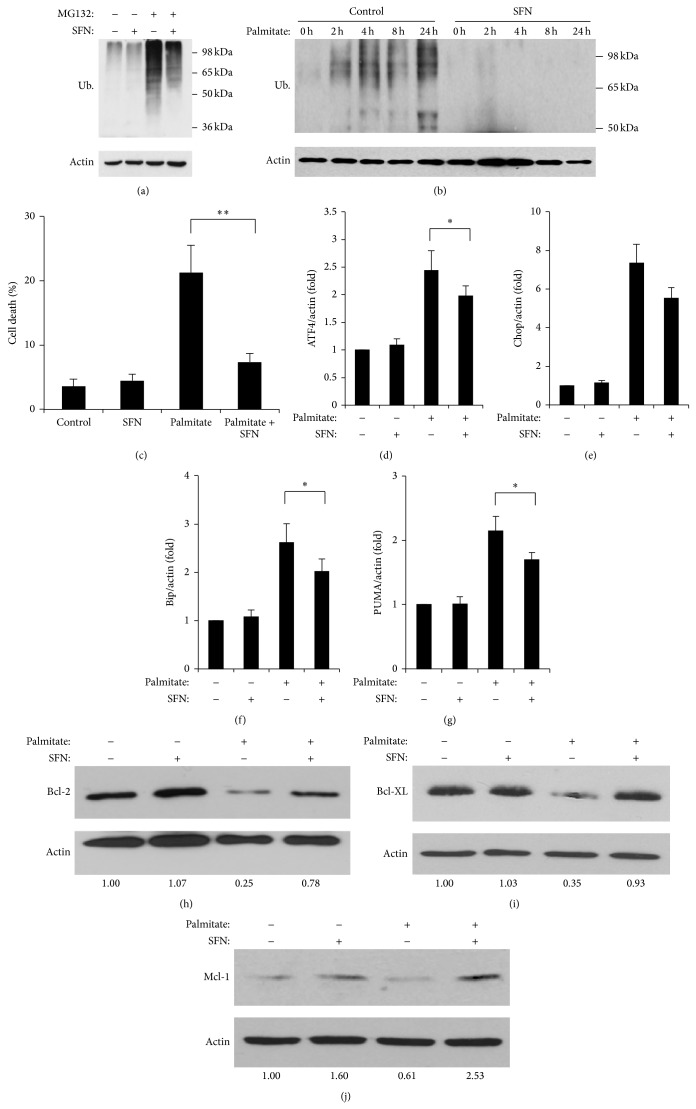
*SFN protects β-cells from FFA-induced cell death.* (a) MIN6 cells were treated with 10 *μ*M MG132 and 2 mM SFN for 24 h and levels of ubiquitinated proteins were measured by Western blot. The result is representative of two independent experiments. (b) MIN6 cells were treated with SFN and palmitate as indicated and Western blot analysis was conducted to detect ubiquitinated proteins. *β*-actin levels were used as a loading control. The result is representative of two independent experiments. (c) MIN6 cells were treated for 24 h as indicated and viability was detected by HO/PI. Results are the means ± SEM of 4 independent experiments. ^∗∗^ *P* < 0.01. ((d)–(g)) MIN6 cells were treated with 0.5 mM palmitate and/or 2 mM SFN for 24 h and ER stress markers (ATF4, Chop, and Bip) and PUMA measured by qPCR as indicated. Results are the means ± SEM of 3-4 independent experiments. ^∗^ *P* < 0.05. ((h)–(j)) MIN6 cells were treated with 0.5 mM palmitate and/or 2 mM SFN for 24 h and expression of prosurvival Bcl-2 proteins was measured by Western blotting with antibodies detecting Bcl-2 (h), Bcl-XL (i), and Mcl-1 (j). Quantification of protein bands is indicated at the bottom as a ratio to *β*-actin loading control. The results are representative of two independent experiments.

In order to determine whether palmitate-induced UPS inactivation can be prevented, we used the proteasome activator SFN. The mechanism of action of SFN involves activation of Hsp27, a heat shock protein that facilitates degradation of ubiquitinated proteins by the proteasome [29]. SFN exposure decreased levels of MG132 induced ubiquitinated proteins (Figure 6(a)). Similarly, SFN also ameliorated the build-up of ubiquitinated protein as a result of palmitate treatment (Figure 6(b)), providing further evidence of the inhibitory effects of palmitate on the UPS. Importantly, palmitate-induced *β*-cell death was prevented by SFN (Figure 6(c)). In line with this result, SFN was able to reduce the levels of palmitate-induced ER stress markers (Figures 6(d)–6(f)), PUMA expression (Figure 6(g)), and prosurvival Bcl-2 protein inactivation (Figures 6(h)–6(j)).

Overall, these results demostrate that activation of the proteasome with SFN can counteract the proapoptotic pathways induced by FFAs and MG132.

### 3.4. Overexpression of Bcl-2 Protein Protects Islets from FFA-Induced Cell Death and Improves *β*-Cell Function during Obesity

**Figure 7:**
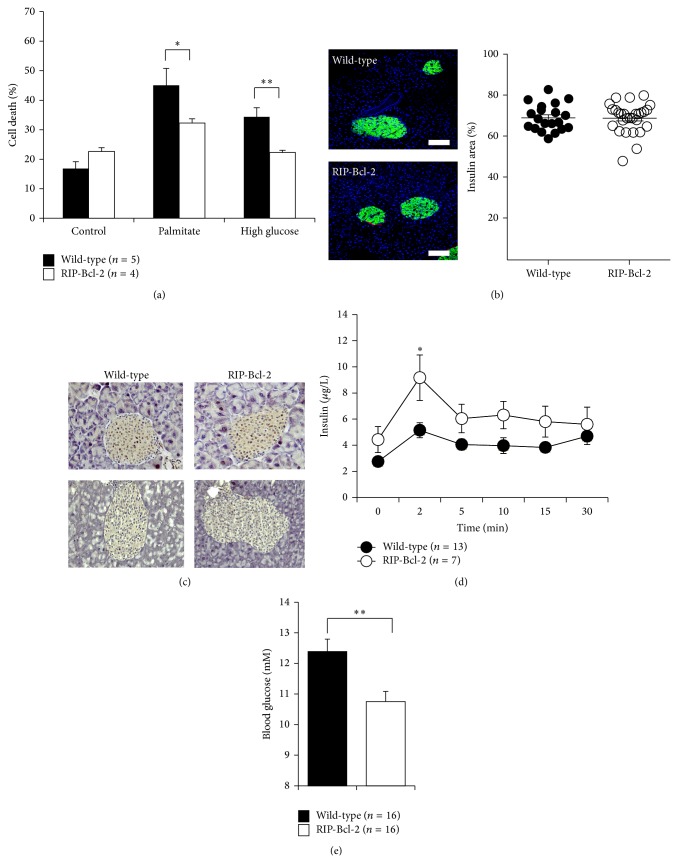
*Overexpression of Bcl-2 improves β-cell function during obesity.* (a) Islets were obtained from wild-type C57BL/6 mice and RIP-Bcl-2 transgenic mice. Islets were then treated with 0.5 mM palmitate or 30 mM D-glucose for 5 days and subsequent levels of cell death were determined by FACS analysis. (b) Pancreas sections from wild-type and RIP-Bcl-2 mice fed on high fat for 16 weeks were stained for insulin (green), and quantification of islets from 5 mice per group is provided as percentage of islet area. BAR, 100 *μ*m. (c) Pancreas sections from wild-type and RIP-Bcl-2 mice fed on high fat for 16 weeks were stained for ubiquitin. (d) IV-GTT results are shown in wild-type and RIP-Bcl-2 high fat fed mice. (e) Fasting (8 h) blood glucose levels of wild-type and RIP-Bcl-2 mice fed on high fat for 16 weeks. ^∗^ *P* < 0.05; ^∗∗^ *P* < 0.01.

Our results suggest that modulation of Bcl-2 protein in response to inactivation of UPS is critical for FFA-induced *β*-cell death. Thus, to restore the imbalance in survival Bcl-2 proteins, we took advantage of the RIP-Bcl-2 transgenic mouse model, in which Bcl-2 is specifically overexpressed in *β*-cells [18]. Bcl-2 overexpressing islets were protected from cell death induced by palmitate (partial protection) or high glucose concentrations (complete protection) (Figure 7(a)). RIP-Bcl-2 mice are viable and do not present any metabolic phenotype on a chow diet (data not shown). RIP-Bcl-2 and wild-type control mice were placed on a high fat diet for 16 weeks. There were no significant differences in incremental body weight, levels of ubiquitinated proteins, and insulin levels between wild-type and RIP-Bcl-2 mice (Figures 7(b) and 7(c) and Supplementary Figure 2). To evaluate whether Bcl-2 overexpression improves *β*-cell function in islets with increased ubiquitinated proteins, we performed an IV-GTT after 14 weeks on high fat diet. Mice overexpressing Bcl-2 secreted enhanced levels of insulin after the glucose challenge indicating improved *β*-cell function (Figure 7(d)). In line with this finding, fasting blood glucose levels were reduced in high fat fed RIP-Bcl-2 mice (Figure 7(e)). In conclusion, transgenic overexpression of Bcl-2 protects islets from cell death caused by obesogenic conditions and improves glucose-induced insulin secretion during obesity.

## 4. Discussion

Previous studies have shown that deregulation of Bcl-2 proteins in pancreatic *β*-cells under conditions associated with type 2 diabetes triggers apoptosis [14, 16]. Here, we provide evidence that FFA-induced inactivation of UPS mediates this modulation of Bcl-2 proteins ultimately resulting in *β*-cell death.

In the present study, we have demonstrated that an increased amount of ubiquitinated proteins in pancreatic islets and in MIN6 cells results from FFA exposure. First, levels of ubiquitinated proteins were increased in obese conditions in humans and mice (Figure 1). Second, proteasome activity was decreased in islets and *β*-cells under lipotoxic stress (Figure 2). Finally, the build-up of ubiquitinated proteins was ameliorated when the proteasome was activated by SFN (Figure 6). The inactivation of UPS in *β*-cells under lipotoxic stress possibly includes transcriptional and posttranscriptional mechanisms. For example, palmitate has been shown to induce upregulation of PSMA7 and PSMC4 genes and downregulation of PSMD10 and PSMB8 genes in human islets [30]. These genes are associated with proteasome function and activity. Moreover, posttranscriptional inactivation of the UPS takes place as a result of increased reactive oxygen species production with FFA oxidation, which distorts the cylindrical structure of the proteasome [31]. Increased saturated fats might also inhibit the activity of enzymes that are associated with the UPS [30, 32]. It is also possible that alternative pathways (e.g., altered activity of U3 ligases) may contribute to palmitate-induced protein ubiquitination.

**Figure 8:**
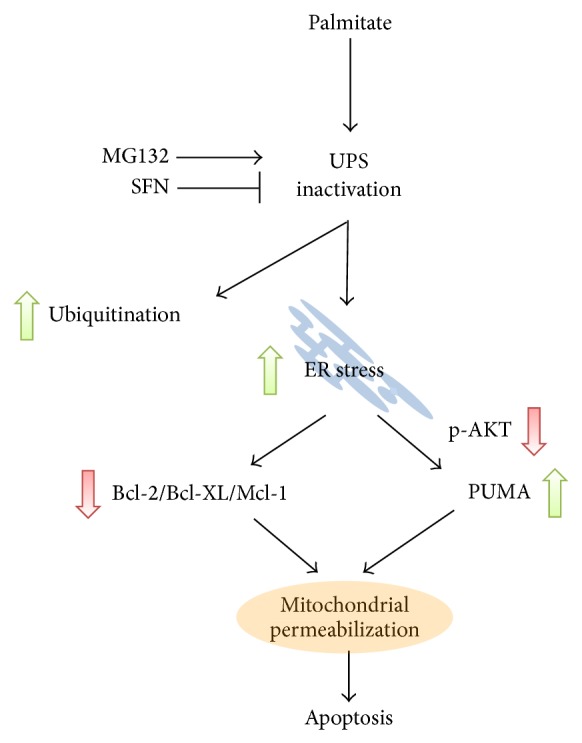
*Proposed model for the role of UPS inactivation in palmitate-induced β-cell apoptosis.* Palmitate induces inactivation of the UPS in *β*-cells resulting in increased ubiquitinated proteins, ER stress, and posttranscriptional inactivation of Bcl-2 and other prosurvival Bcl-2 proteins. In addition, AKT is dephosphorylated leading to upregulated expression of the BH3-only protein PUMA. These signals converge on mitochondrial permeabilization causing *β*-cell demise.

Collectively our data indicate that cell death induced by UPS inactivation and high levels of ubiquitinated proteins is mediated by ER stress and deregulation of Bcl-2 proteins (Figure 8). Specifically, we showed that palmitate increased expression of the ER stress markers ATF4, Chop, and Bip in agreement with previous studies [28]. Lipotoxic ER stress causes the activation of the proapoptotic BH3-only protein PUMA through a complex pathway involving TRB3, AKT, and FoxO3a (present data, [16]). In addition, palmitate decreases prosurvival Bcl-2 protein expression [16, 33, 34]. Here we have demonstrated that decreased Bcl-2 protein expression in palmitate-exposed *β*-cells is dependent on UPS inactivation. Although in this study we focused our attention on UPS inactivation by FFAs, it is probable that alternative factors also contribute to the deleterious effect on type 2 diabetes. Indeed, it has been recently shown that high glucose exposure hypersensitized *β*-cells to apoptosis induced by proteasome inhibitors [35].

It is noteworthy that cell death caused by UPS inactivation was reversed with activation of the proteasome by SFN. The proteasome activator SFN has been suggested in studies as a possible therapeutic treatment for various diabetes conditions such as nephropathy and aortic damage [36, 37]. Given that we have shown that SFN treatment is able to protect *β*-cells from FFA-induced cell death, our findings suggest a potential novel therapeutic target for this aspect of diabetes. The mechanisms of SFN protection in *β*-cells include decreased ubiquitinated proteins, amelioration of ER stress together with PUMA activation, and upregulation of prosurvival Bcl-2 proteins. SFN may also induce *β*-cell protection by inactivation of oxidative stress [38]. It should also be considered that prolonged SFN exposure could attenuate glucose-stimulated insulin secretion in pancreatic *β*-cells [24]. Importantly, we have demonstrated improved *β*-cell function* in vivo* by overexpressing the prosurvival protein Bcl-2. The mechanism of improved insulin secretion in RIP-Bcl-2 mice can be mediated by Bcl-2-induced *β*-cell survival, Ca^2+^ traffic, or glucokinase activity [14], and this will be the subject of future work.

## 5. Conclusions

In conclusion, we found that the FFA palmitate inhibits the UPS, causing *β*-cell apoptosis, and that this effect could be reversed by activation of the proteasome. The mechanism of *β*-cell death involves the deregulation of Bcl-2 proteins via activation of ER stress signaling. In this context, overexpresison of the prosurvival protein Bcl-2 improved *β*-cell function in a mouse model of obesity. Together, our data clarify the mechanism by which FFAs induce *β*-cell death and provide therapeutic targets to improve glucose homeostasis in type 2 diabetes.

## Supplementary Material

The Supplementary Material provides additional information of Figures 1, 3, 7 and Material and Methods.
