# Supplementary material for: Lipotoxic Stress Induces Pancreatic β-Cell Apoptosis through Modulation of Bcl-2 Proteins by the Ubiquitin-Proteasome System
Source: J Diabetes Res. 2015 May 6;2015:280615. doi: 10.1155/2015/280615 (PMC4438180; doi:10.1155/2015/280615)
Supplement: Supplementary file 1 — The Supplementary Material provides additional information of Figures 1, 3, 7 and Material and Methods. [file 280615.f1.pdf]

Supplemental Data

Lipotoxic stress induces pancreatic β-cell apoptosis through modulation of Bcl-2 proteins by the ubiquitin-proteasome system.

Sara A. Litwak, Jibran A. Wali, Evan G. Pappas, Hamdi Saadi, William J. Stanley, L. Chitra Varanasi, Thomas W. C. Kay, Helen E. Thomas, Esteban N. Gurzov

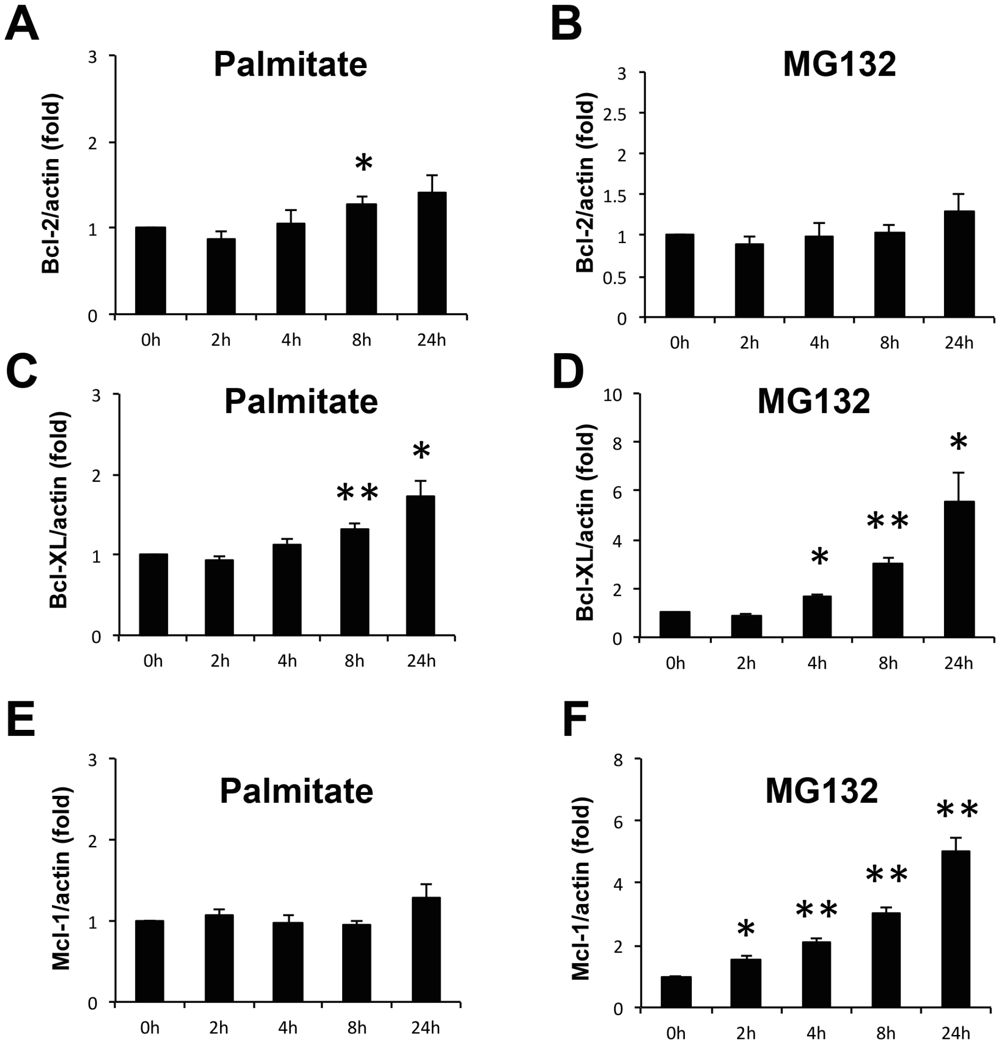

Supplementary Figure 1. Palmitate and MG132 do not reduce mRNA levels of pro-survival proteins in β-cells. (A,C,E) MIN6 cells were treated with 0.5 mM palmitate (A, C, E) or 10 μM MG132 (B, D, F) and expression of pro-survival Bcl-2 (A,B), Bcl-XL (C,D) and Mcl-1 (E,F) was measured by qPCR. Results are the means ± SEM of 3 independent experiments, and represented as fold induction compared to the untreated control samples. *P < 0.05, **P < 0.01.

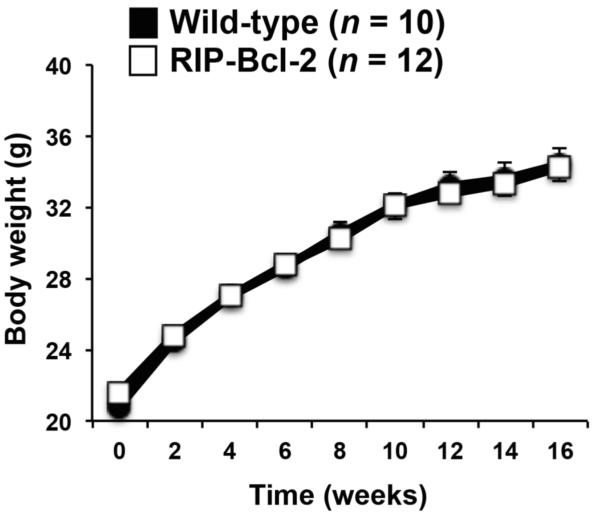

Supplementary Figure 2. Six week-old male wild-type and RIP-Bcl-2 mice were fed a high fat diet for 16 weeks and body weights measured.

Sample ID	Age	Gender	BMI	T2D
SVI-032-10	40	F	24.2	-
SVI-034-10	31	M	21.2	-
SVI-021-10	70	F	22.6	-
SVI-021-11	53	F	34.5	-
SVI-007-11	47	M	38.9	+
SVI-035-10	53	M	30.1	+
SVI-023-14	61	F	32	-
SVI-024-11	47	M	38.9	-

Supplementary Table 1. List of human samples used in the study.

Probe	Catalogue number (Applied Biosystems, Foster City, CA, USA)
**ATF4 (mouse)**	Mm00515325_g1
**Chop (mouse)**	Mm01135937_g1
**Bip (mouse)**	Mm00517690_g1
**Bcl-2 (mouse)**	Mm00477631_m1
**Bcl-XL (mouse)**	Mm00437783_m1
**Mcl-1 (mouse)**	Mm00725832_s1
**PUMA (mouse)**	Mm00519268_m1
**β-actin (mouse)**	Mm00607939_s1

Supplementary Table 2. List of probes used for qPCR. Real-time PCR was performed using the Rotor-Gene RG-3000 machine (Corbett Research; Qiagen, Hilden, Germany) and the TaqMan PCR Master Mix (AmpliTaq Gold with GeneAmp kit; Applied Biosystems) in 20 μl reaction volumes.

Antibody	Company	Reference	Dilution
**Ubiquitin**	Cell Signaling, Danvers, MA	#3933	1/1000
**Cleaved Caspase-3**	Cell Signaling, Danvers, MA	#9661	1/500
**Chop**	Santa Cruz Biotechnology, CA	sc-575	1/500
**Bcl-2**	Cell Signaling, Danvers, MA	#2870	1/1000
**Bcl-XL**	Cell Signaling, Danvers, MA	#2764	1/500
**Mcl-1**	Cell Signaling, Danvers, MA	#5435	1/1000
**β-Actin**	Santa Cruz Biotechnology, CA	sc-7210	1/5000
**HRP-conjugated anti-rabbit IgG**	Millipore, Temecula, CA	12-348	1/5000
**HRP-conjugated anti-mouse IgG**	DAKO, Denmark	P0260	1/10000

Supplementary Table 3. List of antibodies used for Western blot analysis.
